# Quantifying the Socio-Economic Benefits of Reducing Industrial Dietary Trans Fats: Modelling Study

**DOI:** 10.1371/journal.pone.0132524

**Published:** 2015-08-06

**Authors:** Jonathan Pearson-Stuttard, Julia Critchley, Simon Capewell, Martin O’Flaherty

**Affiliations:** 1 Clinical Academic Graduate School, Division of Medical Sciences, University of Oxford, Oxford, United Kingdom; 2 Population Health Research Institute, St George’s, University of London, London, United Kingdom; 3 Division of Public Health and Policy, University of Liverpool, Liverpool, United Kingdom; University of Toronto, CANADA

## Abstract

**Background:**

Coronary Heart Disease (CHD) remains a leading cause of UK mortality, generating a large and unequal burden of disease. Dietary trans fatty acids (TFA) represent a powerful CHD risk factor, yet to be addressed in the UK (approximately 1% daily energy) as successfully as in other nations. Potential outcomes of such measures, including effects upon health inequalities, have not been well quantified. We modelled the potential effects of specific reductions in TFA intake on CHD mortality, CHD related admissions, and effects upon socioeconomic inequalities.

**Methods & Results:**

We extended the previously validated **IMPACTsec** model, to estimate the potential effects of reductions (0.5% & 1% reductions in daily energy) in TFA intake in England and Wales, stratified by age, sex and socioeconomic circumstances. We estimated reductions in expected CHD deaths in 2030 attributable to these two specific reductions. Output measures were deaths prevented or postponed, life years gained and hospital admissions. A 1% reduction in TFA intake energy intake would generate approximately 3,900 (95% confidence interval (CI) 3,300–4,500) fewer deaths, 10,000 (8,800–10,300) (7% total) fewer hospital admissions and 37,000 (30,100–44,700) life years gained. This would also reduce health inequalities, preventing five times as many deaths and gaining six times as many life years in the most deprived quintile compared with the most affluent. A more modest reduction (0.5%) would still yield substantial health gains.

**Conclusions:**

Reducing intake of industrial TFA could substantially decrease CHD mortality and hospital admissions, and gain tens of thousands of life years. Crucially, this policy could also reduce health inequalities. UK strategies should therefore aim to minimise industrial TFA intake.

## Introduction

Coronary Heart Disease (CHD) continues to be a leading cause of mortality and morbidity in the UK. Despite halving of (CHD) mortality rates over the past two decades [1] approximately 35% of total UK deaths are still attributable to Cardiovascular Disease (CVD). Coupled with the chronic disease burden, especially in the older age groups [2], this is estimated to cost the economy £30 billion every year [3] with some £14 billion spent annually on healthcare alone. Major behavioural risk factors for CHD are diet and smoking, followed by excess alcohol consumption and physical inactivity [4]. Within diet the key factors include low intakes of fruit and vegetables, and wholefoods, and an excess intake of salt, sugar, saturated fats, and trans fatty acids (TFA).

TFA consumption comprises industrial TFA (approximately 1% daily energy intake), and ruminant TFA (approximately 0.5% daily energy intake). **Industrial trans fats** are unsaturated fatty acids with at least one double bond in the trans configuration, formed during the partial hydrogenation of vegetable oils. This process is utilised in the production of margarines, commercial cooking and manufacturing processes. The partial hydrogenation process provides a solid fat with longer shelf life, stability during packing, and enhanced palatability. The major sources of TFA in the UK are bakery products, spreads, packaged snack foods and deep-fried fast foods. Naturally occurring TFA, such as in meats, dairy and other ruminant products are produced by the action of bacteria in the ruminant stomach, and account for approximately 0.5% of total energy intake in the UK population. The role of ruminant TFA upon CHD risk is much less well characterised than industrial TFA, and as a recent review has highlighted [5], this topic remains debated, particularly by the dairy industry. Whilst the harmful effect of ruminant TFA was thought to be relatively minor in the past, there is a growing argument that their CHD risk may have been underestimated because of their relatively low intake levels [5].

Industrial TFA substantially increase CHD risk by raising LDL-cholesterol, reducing HDL-cholesterol, causing systemic inflammation and adversely affecting endothelial cell dysfunction [6]. On a per calorie basis, TFA increase CHD risk more than any other macronutrient [6] as every 1% increase in daily energy obtained from TFA raises CHD mortality by 12% [6]: CVD (primary) prevention policies, highlighting reductions in TFA, are therefore gaining more attention and traction. In developed countries, the average daily TFA intake has fallen over the past decade reaching approximately 1.3% (2.8grammes) of daily energy intake in the UK [7]. This fall can be attributed to pressure from UK Foods Standard Agency (FSA) 2003–2007 reformulation efforts, and voluntary content labelling globally [8]. However, this purely voluntary approach appears to be becoming exhausted as the rate of reduction has recently (from 2007–2011) slowed [9]. Yet even such apparently low average levels of TFA have dangerous consequences and increase CHD risk substantially, particularly in deprived groups consuming higher amounts of TFA [10].

Several effective policies have been utilised to successfully reduce dietary TFA across the globe, including voluntary self regulation, content labelling, or local or national legislation [11]. New York City led the US through reduction in TFA in restaurant food and voluntary self regulation; this has seen population TFA levels halve in the USA [9]. However, due to incomplete population coverage, this approach allows residual pockets of the population to have a persistently high TFA intake [11] due to more deprived communities tending to purchase more processed products which provide the ‘cheap’ calories and meals. National legislative bans, such as seen in Demark, Iceland, Austria and Switzerland have been the most effective policies, essentially eliminating industrial TFA in foods [12]. Achieving the lowest possible TFA level is therefore of great importance. There is no room for complacency, and considerable scope remains to develop further policies to continue this reduction within the UK population.

Furthermore, UK health inequalities in socio-economic circumstances (SEC) remain substantial [13]. Thus, despite the one third reduction in CHD mortality between 1980–2007, large inequalities have persisted, and even worsened in some age groups [14]. Treatment uptake appears surprisingly equitable across SEC quintiles [15] clearly suggesting that much of the persistent CHD inequality must be attributable to differences in major cardiovascular risk factors [16]. However, in order to implement more effective prevention policies in the future, UK policy makers will require solid evidence to assess and quantify the potential benefits in tackling CHD inequalities achievable through population level reductions of TFA.

We therefore aimed to quantify the potential benefits of population level reductions in TFA consumption in the UK. Further, we aimed to quantify the effects upon inequalities in mortality, life years and hospital admissions, in order to help inform future prevention strategies.

## Methods

### Data Sources for the IMPACT CHD Model

We extended the current and validated IMPACT_SEC_ CHD model calibrated for the English and Welsh population to estimate the effect of different population level reductions in TFA on mortality, life-years, and the underlying CHD burden. We named this IMPACT_TFA_ model. The population was stratified by age (10 year age groups from 25–34 up to 85+), gender and SEC (quintiles based on Index of Multiple Deprivation (IMD) scores). The Index of Multiple Deprivation (IMD) is a composite index of relative deprivation at small area level based on seven domains: income; employment; health deprivation and disability; education, skills and training; barriers to housing and services; crime and disorder; and living environment [17]. The age groups were then further categorised to analyse trends in young, middle aged, and elderly (less than 55 years, 55 to 74 years, and ≥ 75 years old). Mortality and demographic data for the IMPACT_SEC_ model were obtained from the Office of National Statistics. Patient numbers were estimated for seven mutually exclusive patient groups (Acute Myocardial Infarction (AMI), unstable angina (UA), secondary prevention post AMI, secondary prevention post revascularisation, angina in the community, heart failure admissions, heart failure in the community) using data from Hospital Episodes Statistics, Myocardial Ischaemia National Audit Project and General Practice Research Database. For the TFA model we only included incidence patient numbers, ie AMI, UA and heart failure admissions due to insufficient data regarding TFA affect upon case fatality. The mortality reduction for a given reduction in TFA was taken from the systematic review by Mozaffarian (2006) [6], whilst this effect was stratified into age and sex specific reductions in mortality by O’Flaherty et al [10], assuming that they are consistent with the age and gender coronary heart disease mortality effects gradients observed with cholesterol changes [18]. Upper and lower estimates for the probabilistic sensitivity analysis were generated using 80% and 120% of the mean estimates respectively. This effect size is for total effect of TFA upon CHD mortality, rather than through cholesterol effects alone. The effect gradient across these groups was conserved to create mortality reduction factors for given levels of reductions in TFA intake resulting in TFA intake of 0.5% and 0% of daily energy intake respectively. Stratifications, by age and gender, of mortality reduction factors, for a 1% reduction, and 0.5% reduction in trans fats intake (as a percentage of daily energy intake) are available in table B in S1 File. As no data was available from this systematic review among the oldest age group (≥85), a mortality reduction was calculated by extrapolation from the younger age groups and tested in a probabilistic sensitivity analysis. The mortality reduction among those ≥ 85 was extrapolated by assuming that the same attenuation of the relative risk would occur with age as has been found for the relationship between total cholesterol and CHD mortality risk by age.

#### IMPACT_TFA_ Model Methods

The expected number of deaths in 2016, 2020 and 2030 were calculated by using a Bayesian Age-period-cohort model based forecast [19]. To estimate the effect of changes in TFA intake, we calculated the number of expected deaths from CHD occurring at each time point (2016, 2020 and 2030) first with no change in trans fats intake and second assuming a certain reduction in TFA intake in the corresponding year (2016, 2020 and 2030). Using the mortality reduction values for given levels of TFA reduction as described above assuming a linear dose-response relationship between TFA intake and CHD risk, this provided the deaths prevented or postponed (DPPs) for men and women in each age and socio-economic circumstance quintile.

Using the calculated DPPs, we then estimated the number of life-years gained by multiplying the deaths prevented or postponed for the specific reduction in TFA by the age specific median survival for the different population subgroups (diagnosed CHD, undiagnosed CHD and population free of CHD). Estimates of median survival for these subgroups were initially obtained from a previous analysis performed for the England and Wales population for 2000 [20–23], and was updated with more recent (2010) data adapted from Smolina et al [24]. Here, median survival data was obtained from the best available population-based data for those patients with recognised CHD, symptomatic but unrecognised CHD, and asymptomatic individuals. We used probabilistic sensitivity analysis for parameter uncertainty. This was done using Monte Carlo simulation, involving repeating the estimation of the model, drawing parameter values from their respective statistical distributions. The distributions and sources of each input into the sensitivity analysis are outlined in table A in S1 File. We then used the Microsoft Excel add-in program Ersatz software to perform 5,000 simulations to determine the 95% confidence intervals of the DPPs (2.5^th^ and 97.5^th^ percentile values corresponding to lower and upper limits). For simplicity, rounded ‘best estimates’ and precise confidence intervals are included in the text, whilst precise values are presented in the subsequent tables and figures. The lower and upper confidence intervals are derived from 5% and 95% centiles of 10,000 Monte Carlo simulations. Since many input variables are not normally distributed the confidence intervals may not be symmetrical.

The resulting effect of given reductions in TFA upon the CHD burden was derived from the incidence patient numbers. This included hospital admissions for acute myocardial infarction, unstable angina and heart failure and excluded community prevalence numbers of angina, heart failure and myocardial survivors living in the community. This was due to an assumed negligible effect of this policy upon case fatality itself, hence limited effect upon underlying community prevalence. The resulting number of patients were derived from the mortality reduction percentage and existing incidence numbers by disease group. The relative distribution and ratios of patients across the disease groups within the underlying burden remained constant from the initial patient numbers to the resultant.

### Socio-economic circumstance analysis

The SEC component of the IMPACT model used the Index of Multiple Deprivation (IMD) as previously described [14]. This is a widely used measure in the UK of relative area deprivation based on seven domains: income; employment; health deprivation and disability; education; skills and training; barriers to housing and services; crime and disorder; and living environment [17]. To model the effects of given reductions in TFAs upon the health inequalities within the CHD burden, we modelled two different scenarios. Firstly, a conservative model, which assumed equal TFA intake as a percentage of daily energy intake across SECs, named TFAsec1. Secondly, a model assuming an unequal intake of TFA as a percentage of daily energy intake across SEC quintiles (table B in S1 File), named TFAsec2. No adjustment for total daily energy intake by SEC was made. These models stratified the data by socio-economic circumstance in the following ways:

1. The UK coronary heart disease population are stratified into SEC IMD quintile populations
2. SEC quintile specific mortality counterfactuals
3. SEC quintile specific median survivals
4. SEC quintile specific intake of TFA in TFAsec2

SEC specific mortality counterfactuals were calculated for the year 2030, whilst median survival for the ‘undiagnosed CHD’ and ‘no CHD’ groups were stratified by SEC. This was done using life expectancies by SEC IMD quintiles from 2007–10 [25] and creating indices of life expectancy where SEC quintile three was the index, 1.0. The respective index for each SEC quintile was then used to calculate a more specific median survival by SEC. In the case of the ‘undiagnosed CHD’ group, median survival was taken as the midpoint between the ‘diagnosed CHD’ and ‘no CHD’ groups. The mean TFA intake across the UK population was taken from the Low Income Diet and Nutrition Survey [7] (1.3%) whilst tailored trans fats intake by SEC quintile was adapted from this survey. This is outlined in table B in S1 file.

In TFAsec1, whereby TFA intake is assumed to be equal across SEC quintiles, the results are additionally presented as an index, using socio-economic circumstance quintile 3 as the index 1.0. Further, these indices are calculated using percentages, with the denominator being the specific UK population for that SEC quintile, age and sex group. This was done to use rates, rather than crude numbers to avoid misleading results due to the large differences in crude population numbers across SEC quintiles in the older age groups, owing to variation in their respective life expectancies.

## Results

### Effects of a reduction in trans fatty acids intake by 1% and 0.5% (as % of daily energy intake) on deaths prevented or postponed, life years gained and Hospital Admissions

#### Deaths Prevented or Postponed

**Fig 1:**
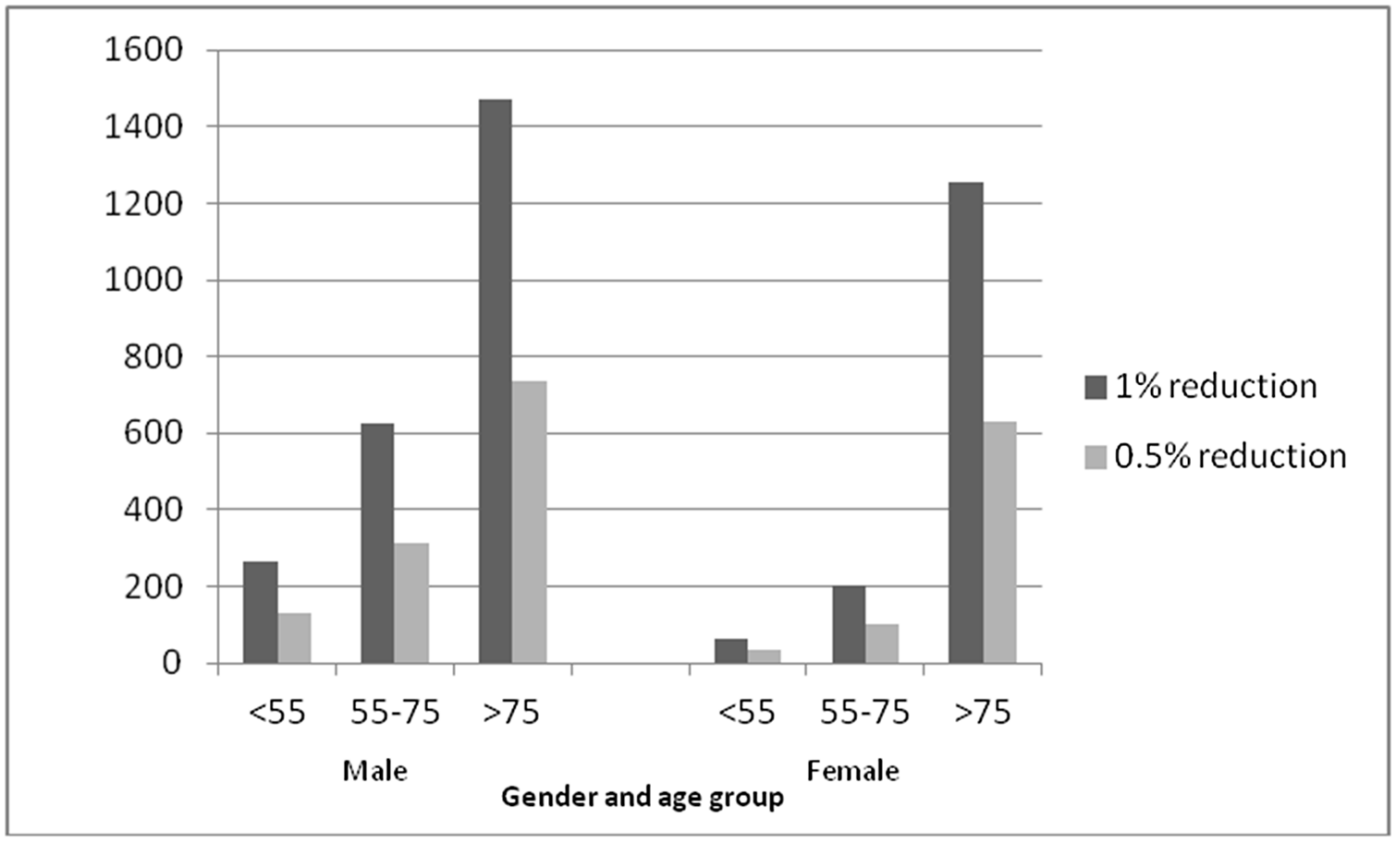
Deaths prevented or postponed (DPPs) per year with a 1% and 0.5% reduction in daily energy intake of trans fatty acids intake. DPPs by age and sex. *Data source*: *Hospital Episode Statistics*.

A 1% reduction in TFA of daily energy intake across the England and Wales population would result in approximately 3,900 (95% confidence interval (CI) 3,325–4,453) DPPs per year. Over half (60%, n = 2400) of these deaths prevented would be among men, with the remainder (n = 1500) among women; and most among people over 75 years old. (Table 1, Fig 1)

**Table 1:** The effects, per year, upon the UK population of a reduction from 1% to 0% in trans fatty acids of daily energy intake. *Numbers of Deaths prevented or postponed (DPP)*, *life years gained (LYG)*, *Reductions in Acute Myocardial Infarction (AMI) Admissions*, *Reductions in Unstable Angina (UA) Admissions and reductions in Heart Failure (HF) admissions*. *Stratified by age and gender*. *Reduction in TFA intake by 0*.*5% daily energy yield half of the below gains*.

TFA					Age		
1% reduction	Totals	95% CI	95% CI	<55		55–75	>75
**DPP**	**3900**	*3325*	*4453*				
Men	2400			265		625	1471
Women	1500			64		203	1257
**LYG**	**37000**	*30106*	*44670*				
Men	20000			6721		7012	6014
Women	17000			2381		4198	10718
**AMI admissions reductions**	**1500**	*1320*	*1761*				
Men	1000			359		431	237
Women	500			82		169	263
**Unstable Angina admissions reductions**	**6800**	*5865*	*7828*				
Men	4100			1442		1807	823
Women	2600			564		972	1102
**Heart Failure admissions reductions**	**1500**	*1325*	*1766*				
Men	800			90		290	430
Women	700			46		166	523

The lower and upper confidence intervals are derived from 5% and 95% centiles of 10,000 Monte Carlo simulations. Since many input variables are not normally distributed the CI may not be symmetrical. All figures above are per annum. Totals are rounded.

A 0.5% reduction in TFA of daily energy intake would result in approximately 1900 (95% CI: 1,660–2,228) deaths prevented or postponed per year. (Fig 1)

#### Life Years Gained

**Fig 2:**
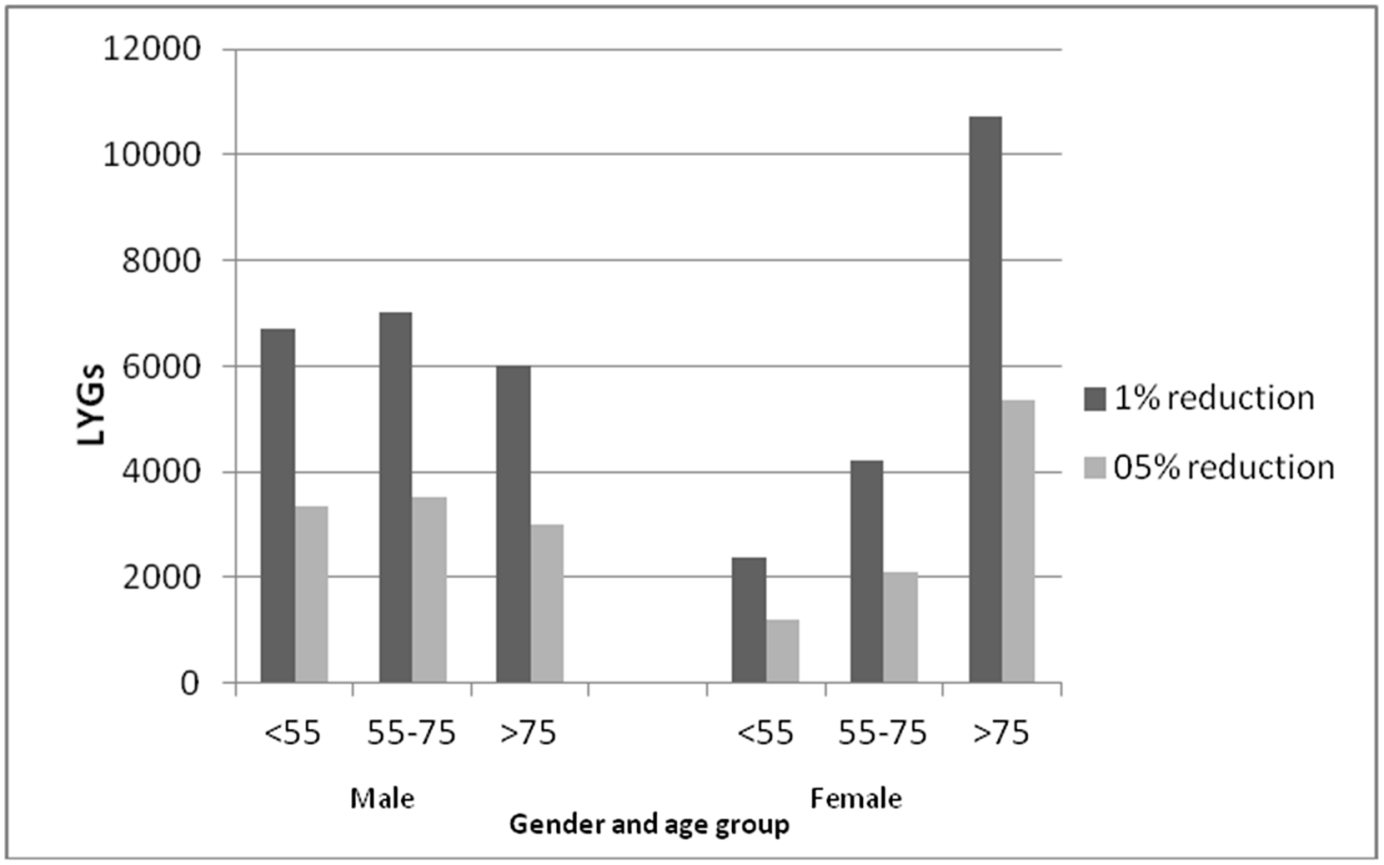
Life years gained (LYG) per year with a 1% and 0.5% reduction in daily energy intake of trans fatty acids. Life years gained (LYG) by age and sex. *Data source*: *Hospital Episode Statistics*.

A 1% reduction in TFA of daily energy intake across the England and Wales population would result in some 37,000 (95% CI: 30,106–44,670) LYGs per year. About 20,000 of these would be among men, and 20,000 among women). Although most LYGs would be among the elderly (people over 75), some 25% would be among younger people (under 55 years). (Table 1, Fig 2)

A 0.5% reduction in TFA of daily energy intake would result in approximately 19,000 (95% CI: 15,039–22,309) LYGs per year. (Fig 2)

#### Hospital Admissions

**Fig 3:**
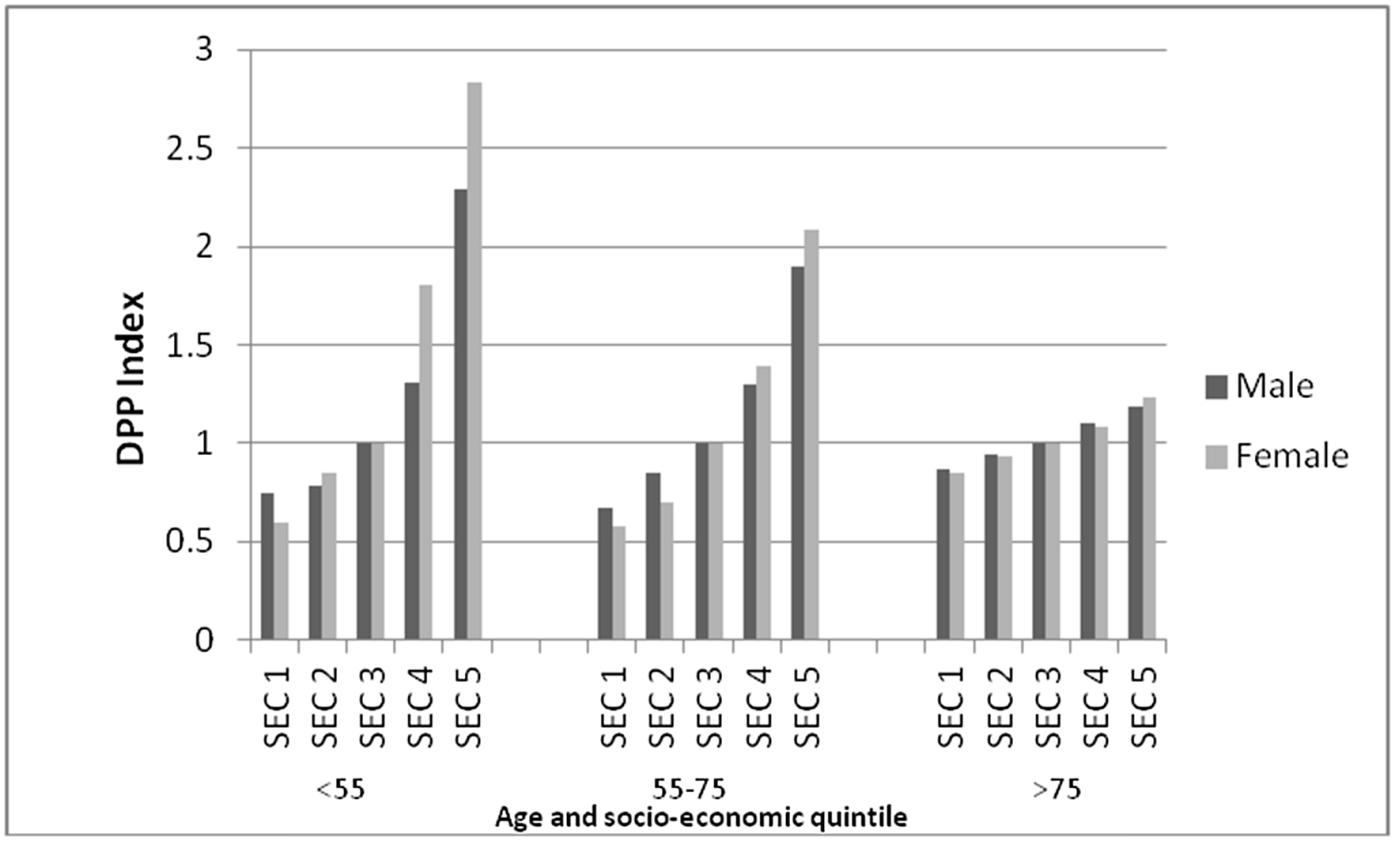
Hospital Admissions of Acute Myocardial Infarction (AMI), Unstable Angina (UA) and Heart Failure (HF) per year with a 1% reduction in daily energy intake of trans fatty acids intake. 4a Male, 4b Female. Hospital admissions by age. *Data source*: *Hospital Episode Statistics*.

A 1% energy reduction in TFA intake would result in approximately 9,800 (95% CI: 8,783–10,258) fewer CHD related hospital admissions than in 2007. This total comprises some 1,500 (95% CI: 1,321–1,761) fewer acute myocardial infarction admissions, approximately 1,500 (95% CI: 1,325–1,766) fewer heart failure (HF) admissions, and some 6,700 (95% CI: 6,139–6,731) fewer unstable angina hospital admissions. This represents a 7% reduction in CHD related hospital admissions. The reductions seen in male hospital admissions (6,000) was greater than for females (4,000), with middle aged men (55–75 years old) seeing the largest potential reductions in unstable angina and acute myocardial infarction admissions. Heart Failure admissions however were reduced most in the oldest age groups (over 75 years). (Table 1, Fig 3)

A small reduction in TFA intake of 0.5% energy would still result in significant health gains. Namely approximately 4,900 (95% CI: 4,256–5,682) fewer CHD related hospital admissions than in 2007. This comprises approximately 750 (95% CI: 660–881) fewer acute myocardial infarction admissions, 750 (95% CI: 663–883) fewer heart failure admissions and 3,400 (95% CI: 2,933–3,917) fewer unstable angina admissions. (Fig A in S1 File)

### The effect upon Coronary Heart Disease socio-economic inequalities

#### Assuming equal intake of trans fatty acids across socio-economic circumstance quintiles

A 1% reduction in TFA intake across all SEC quintiles, conservatively assuming equal intake across SEC quintiles would result in approximately 33% more DPPs in the most deprived quintile than the most affluent. Further, this would also lead to approximately 12,000 (95% CI: 7,447–16,756)LYGs in the most deprived quintile, compared to some 4,000 (95% CI 2,723–6,126) LYGs in the most affluent quintile (Table 2), whilst a 60% greater reduction in hospital admissions would be seen in the most deprived compared with the most affluent group. (Table 3, Fig 3)

**Table 2:** The effects, per year, upon the UK population of a reduction from 1% to 0% trans fatty acids of daily energy intake across all socio-economic circumstance quintiles. *Numbers of Deaths prevented or postponed (DPP)*, *life years gained (LYG)*, *Reductions in Acute Myocardial Infarction (AMI) Admissions*, *Reductions in Unstable Angina (UA) Admissions and reductions in Heart Failure (HF) admissions*. *Stratified by gender and socio-economic circumstance (SEC) quintile*. *0*.*5% reduction in TFA intake yields half below gains*.

TFAsec									
1% reduction	Totals	95% CI	95% CI	SEC1	SEC2	SEC3	SEC4	SEC5	Total
**DPP**	**4000**	*3232*	*4849*						
Men	2500			413	475	503	512	556	2458
Women	1600			261	307	329	340	345	1582
**LYG**	**43000**	*27246*	*61303*						
Men	21700			1180	4206	4669	5198	6439	21691
Women	21400			3258	3825	4283	4708	5282	21357
**AMI admissions reductions**	**3900**	*3082*	*4623*						
Men	2600			448	501	529	531	560	2570
Women	1300			209	247	255	274	298	1283
**Unstable Angina admissions reductions**	**4500**	*3629*	*5443*						
Men	2700			396	457	518	577	718	2666
Women	1900			256	322	356	418	517	1869
**Heart Failure admissions reductions**	**2200**	*1790*	*2685*						
Men	600			80	102	121	140	189	631
Women	1600			269	318	334	338	347	1607

**Table 3:** The effects, per year, upon the UK population of reducing trans fatty acids intake by 1% of daily energy intake across all socio-economic circumstance quintiles. *Numbers of Deaths prevented or postponed (DPP)*, *life years gained (LYG)*, *Reductions in Acute Myocardial Infarction (AMI) Admissions*, *Reductions in Unstable Angina (UA) Admissions and reductions in Heart Failure (HF) admissions*. *Stratified by gender and socio-economic circumstance (SEC) quintile*.

**TFAsec**				**<55yrs**			
**1% reduction**	**Totals**	**SEC1**	**SEC2**	**SEC3**	**SEC4**	**SEC5**	**Total**
**DPP**	400						
Men	300	34	41	53	71	108	308
Women	100	8	10	13	21	34	86
**LYG**	12000						
Men	8000	524	1198	1549	2046	3030	8348
Women	4000	365	435	553	903	1375	3631
**AMI admissions reductions**	1100						
Men	900	130	146	179	198	244	898
Women	200	22	30	33	50	69	204
**Unstable Angina admissions reductions**	1300						
Men	900	107	123	163	209	301	903
Women	400	40	59	69	106	169	442
**Heart Failure admissions reductions**	700						
Men	100	11	12	16	20	31	90
Women	600	85	105	114	122	136	562
				**55-74yrs**			
**1% reduction**	**Totals**	**SEC1**	**SEC2**	**SEC3**	**SEC4**	**SEC5**	**Total**
**DPP**	900						
Men	700	96	120	132	145	181	674
Women	200	28	34	43	51	66	222
**LYG**	11400						
Men	6700	269	1372	1478	1585	1952	6656
Women	4700	611	736	937	1094	1364	4742
**AMI admissions reductions**	1500						
Men	1100	197	226	219	220	215	1078
Women	400	65	79	84	87	106	422
**Unstable Angina admissions reductions**	1900						
Men	1200	176	213	227	248	297	1161
Women	700	98	123	141	157	200	719
**Heart Failure admissions reductions**	800						
Men	200	23	31	37	43	60	194
Women	600	104	122	128	127	129	610
				**_>75yrs**			
**1% reduction**	**Totals**	**SEC1**	**SEC2**	**SEC3**	**SEC4**	**SEC5**	**Total**
**DPP**	2800						
Men	1500	283	314	317	296	266	1477
Women	1300	225	263	274	268	246	1275
**LYG**	15900						
Men	5100	281	1299	1293	1205	1061	5140
Women	10700	1926	2234	2323	2223	2028	10734
**AMI admissions reductions**	800						
Men	600	121	129	131	113	100	594
Women	200	22	30	33	50	69	204
**Unstable Angina admissions reductions**	1300						
Men	600	113	121	128	120	120	603
Women	700	118	141	146	155	148	709
**Heart Failure admissions reductions**	800						
Men	300	46	59	68	76	98	347
Women	400	80	91	92	89	83	435

All figures above are per annum. Totals are rounded.

#### Modelling unequal intake of trans fatty acids across socio-economic circumstance quintiles

**Fig 4:**
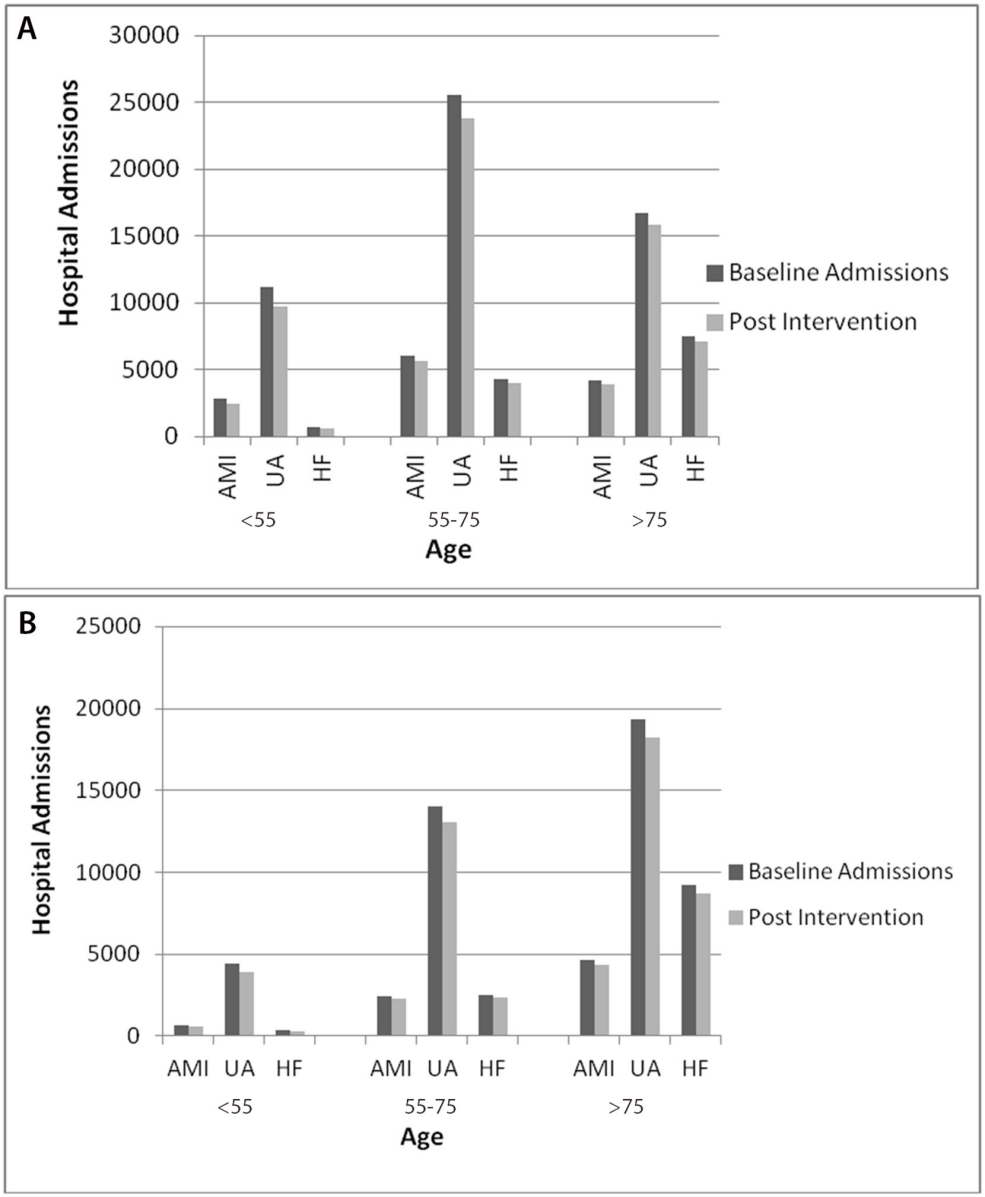
Deaths prevented or postponed (DPPs) Index with a 1% reduction in daily energy intake of trans fatty acids intake. DPPs by age, gender and socio-economic circumstance assuming equal TF intake. *Data source*: *Hospital Episode Statistics*.

**Fig 5:**
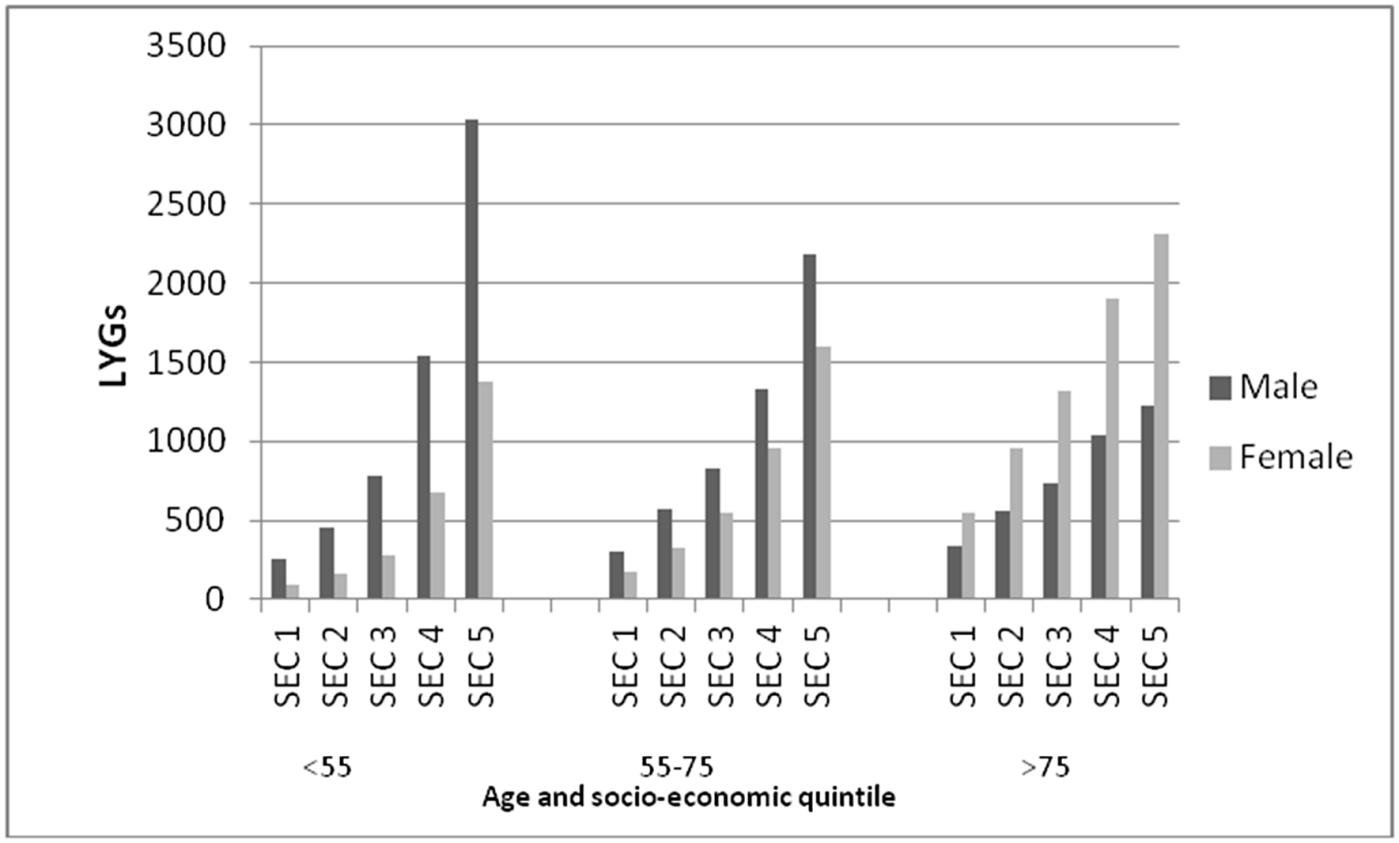
Life Years Gained (LYGs) per year, with a trans-fats daily energy intake of 0.5% across all socio-economic circumstance (SEC) quintiles modelling unequal intake of trans fatty acids across socio-economic circumstance quintiles. LYGs by age, gender and socio-economic circumstance. *Data source*: *Hospital Episode Statistics*.

Reducing dietary TFA intake to 0.5% of daily energy intake (ie eliminating industrial trans fats intake) throughout all SEC groups, would result in approximately 2,400 (95% CI: 1,934–2,901) deaths prevented or postponed (Fig 4), some 28,000 (95% CI: 19,937–37,224) life years gained (Fig 5) and approximately 6,000 (95% CI: 4,808–7,212) fewer hospital admissions. The latter would comprise some 2,300 (95% CI: 1,807–2,711) fewer acute myocardial infarction admissions, approximately 2,800 (95% CI: 2,259–3,389) fewer unstable angina admissions and some 900 (95% CI: 742–1,113) fewer heart failure admissions. This models a baseline of unequal intake of TFA across SEC quintiles. (Fig B in S1 File)

Furthermore, reducing TFA energy intake to 0.5% across all quintiles would result in a five-fold or even six fold greater gains in the most deprived compared with the most affluent quintiles in terms of DPPs, LYGs and hospital admissions avoided (Fig B in S1 File)

## Discussion

Reducing industrial TFA intake in the UK could substantially decrease CHD mortality and hospital admissions whilst gaining tens of thousands of life years. Whilst mortality reductions would be observed primarily in the older age groups, middle aged males would see the greatest reductions in hospital admissions and a significant minority of life years gained would occur among younger people.

### Socioeconomic Circumstances

Reducing TFA intake could also substantially reduce existing health inequalities in CHD [14]. The conservative model, assuming a consistent 1% reduction in TFA intake across all SEC quintiles demonstrated a third more deaths prevented, and a two thirds greater reduction in hospital admissions in the most deprived quintile compared with the most affluent.

However the Low Income Diet and Nutrition Survey (LIDNS) UK survey data [7] demonstrates a very unequal consumption of TFA across SEC quintiles, with the most deprived groups having much higher intake of ‘cheap calories’ such as processed foods and ‘takeaways’. Modelling this unequal intake across SEC quintiles, (assuming a greater fall from higher starting levels in the most deprived groups) (quintile 1–0.75%, quintile 2–0.87%, quintile 3–1%, quintile 4–1.25%, quintile 5–1.5%) in line with LIDNS data suggested even greater potential reductions of the CHD inequalities.

A legislative ban eliminating industrial TFA, as has been achieved in other European countries such as Denmark, would achieve a TFA reduction averaging approximately 0.5% of daily energy intake across all SEC quintiles. This scenario could result in approximately 2,400 fewer deaths, 6,000 fewer hospital admissions and 28,000 LYGs.

Treatment uptake for CHD has been shown to be surprisingly equitable across all socio-economic circumstance quintiles [15], however large SEC gradients in hospital admissions and community prevalence of CHD persist. This suggests that population wide changes in CHD risk factors, which are unequal across SEC quintiles, represent an underutilised tool to address health inequalities in CHD. Our model suggests that population level primary prevention achieving a universal reduction in TFA intake could generate benefits five or six-fold greater in the most deprived quintiles, thus resulting in large reductions in CHD inequalities.

CHD mortality rates fell by approximately 35% between 1999–2007, whilst the overall burden of CHD encompassing hospital incidence and community prevalence fell from 1.9million in 1999 to 1.7 million in 2007 [14]. However, SEC inequalities persisted and even worsened in some age groups during this period. This highlights the inadequacy of previous prevention policies. The case for more effective policies in reducing CHD inequalities has therefore never been stronger. Furthermore, diabetes and obesity, two significant risk factors for CHD, both of which are affected by the intake of TFAtrans fats worsened across all SEC quintiles, but particularly in the most deprived [26].

Other European countries have utilised effective and powerful population-wide policies, to achieve substantial reductions in tobacco, dietary salt, saturated fats, sugars and trans fats. Namely, effective bans on industrial TFA has been achieved in Denmark, Iceland, Sweden and Switzerland, whilst TFA have not been classified ‘Generally Recognised As Safe’ (GRAS) by the FDA, paving the way for future regulation. Such policies have resulted in large and rapid reductions in cardiovascular mortality and morbidity. [27–29]

Our results are reassuringly consistent with previous studies. Mozaffarian et al [30] analysed TFA consumption in Iran, and the effects upon CHD. Baseline intake of TFA was 5–6 times above effective industrial TFA elimination ie 0.5% daily energy intake, than in the UK, whilst baseline mortality rates were also higher. This study suggested that elimination of industrial TFA could prevent 5,600–27,300 CHD deaths. Similarly, Danaei et al [31] suggested that approximately 82,000 CHD events (not deaths) could be prevented if TFA were eliminated in the USA. This study used 2005 TFA intake (2.6% of daily energy) data as baseline, hence our study uses considerably more recent (lower) TFA intake data, giving more modest, albeit still highly significant health benefits. These modelling results are consistent with the significant (50%) reductions in CHD mortality observed in Denmark where TFA intake has been reduced from 6g per day to 1g per day [12].

These results have major implications for both policy and future research. The policy implications include 1) significant health benefits to the UK population might be predicted if industrial TFA intake was reduced across the UK population; 2) such reductions in trans fats intake might also be predicted to substantially reduce existing health inequalities amongst CHD patients within the UK, an issue that has failed to be comprehensively addressed over the past two decades despite being repeatedly highlighted as a priority. The research implications include the IMPACT_TFA_ model demonstrating the utility of modelling primary prevention strategies to analyse potential health benefits across the UK population. This model could be further developed to contain an economic cost-benefit analysis of such potential policies, whilst also comparing the health and economic costs and benefits of different population wide policies. Further, a longer term effect of such policies upon community prevalence could be modelled using a Markov approach.

This modelling study has several strengths, modelling using large data sets which cover the entire adult population of 35 million. Similarly, data quality is generally very good. [16,17,26, 32,33] This is the first study to quantify the effects upon the UK population of a population wide reduction in TFA intake, and importantly the subsequent effect of such policies upon the health inequalities seen in CHD mortality and the underlying burden. This provides quantitative data demonstrating the potential strength of population level approaches to tackling CHD inequalities; a challenge that has made very little progress over the past two decades, despite being highlighted as a priority. The datasets used are representative of the socio-economic distribution of the English and Welsh population, and using such a large dataset allows relatively precise estimates of such policies, allowing conclusions to be drawn to better inform policy makers.

This study also has limitations. We used an area level categorisation of SEC (IMD). This may therefore be sub-optimal for analysing trends within individuals. However, area deprivation measures generally correlate well with measures of individual socioeconomic position in the UK [32]. The age and sex mortality reductions for given levels of TFA reductions were taken from the study by O’Flaherty et al [10]. However the value for the 85+ age group was extrapolated using the reducing mortality reduction figures from the younger age groups. Similarly, the mortality reduction for a 0.25% reduction in trans fat intake was estimated using linear extrapolation. The mortality reductions themselves assumed the same age gradients as cholesterol and similarity between sexes. In addition, the model assumes the effects of the reductions in trans fats intake to be almost instantaneous, whilst this model does not account for how such a reduction, or ban would be implemented. Future work could model and compare various implementation strategies. Further we recognise that upon eliminating TFA, a substitute fat may be used in its place, itself carrying a residual, albeit much lower, CHD risk. We did not formally account for any substitutional effect. Furthermore, we model given reductions in total TFA intake, whereby the majority of the reduction would derive from industrial TFA. We assumed that elimination of ruminant TFA would not be feasible. Moreover, current evidence is insufficient to quantify the effect upon CHD risk of reductions in specifically, ruminant TFA.

Our model uses health and TFA intake data from 2007. However, latest National Diet and Nutrition Survey data suggests that the mean TFA intake has fallen from 1.3% to 0.8% [34]. This suggests that the more modest modelled scenario of a reduction of TFA intake by 0.5% of daily energy, would be more applicable, however, this more modest reduction, could still yield significant health gains, and perhaps more importantly, have a substantial impact upon existing health inequalities within CHD. This further highlights the importance of attaining the lowest TFA intake possible, thus continuing successful reductions over the past decade.

CHD treatment uptake has been shown to be equitable across SEC quintiles [15], however, median survival was not stratified by SEC quintile for patients with CHD. This may have therefore underestimated the true socio-economic gradient in median survival. Furthermore, when modelling the effect upon resulting patient numbers and resulting hospital admission, we assumed no future decline in case fatality. This is clearly a conservative estimate when modelling a 2030 scenario. The real effects upon the underlying CHD burden are thus likely to be larger than those estimated here. The hospital admissions numbers reported as baseline, using diverse data sources may still be an underestimate by failing to capture all readmissions during the same calendar year. This would in turn underestimate the estimated benefits.

### Conclusions

These results provide quantitative data to better inform policy makers. Despite recent substantial reductions, CHD remains a leading cause of mortality and morbidity. It generates a healthcare and economic burden on the increasingly strained UK clinical services. Past efforts to reduce CHD inequalities across socio-economic circumstance quintiles have been unrewarding. In contrast, these results demonstrate that potentially large reductions in CHD mortality, admissions and inequalities might be achieved by any UK reduction in industrial TFA intake. Such population based prevention policies currently remain underused, generating an avoidable and unequal burden on the health services, and on society.

## Supporting Information

S1 File Text A in S1 File, IMPACT England and Wales Trans Fats (IMPACTTFA) extension model. Table A in S1 File, Probability distributions for the IMPACT England and Wales Trans Fats (IMAPCTTFA) Model parameters. Table B in S1 File, Trans fatty acids intake (as a % of daily energy) by socio-economic circumstance (SEC) quintile. Table C in S1 File, Mortality reduction factors for reduction in Trans fats intake (as a % of daily energy) of 1% and 0.5%, stratified by age, and gender. Fig A in S1 File, Hospital Admissions of Acute Myocardial Infarction (AMI), Unstable Angina (UA) and Heart Failure (HF) with a 0.5% reduction in daily energy intake of trans fatty acids intake. Fig B in S1 File Deaths prevented or postponed (DPPs) with a trans fatty acids daily energy intake of 0.5% across all socio-economic quintiles. Strobe Statement A in S1 File.
(DOC)
