# Supplementary material for: Quantifying the Socio-Economic Benefits of Reducing Industrial Dietary Trans Fats: Modelling Study
Source: PLoS One. 2015 Aug 6;10(8):e0132524. doi: 10.1371/journal.pone.0132524 (PMC4527777; doi:10.1371/journal.pone.0132524)
Supplement: S1 File — Text A in S1 File, IMPACT England and Wales Trans Fats (IMPACTTFA) extension model. Table A in S1 File, Probability distributions for the IMPACT England and Wales Trans Fats (IMAPCTTFA) Model parameters. Table B in S1 File, Trans fatty acids intake (as a % of daily energy) by socio-economic circumstance (SEC) quintile. Table C in S1 File, Mortality reduction factors for reduction in Trans fats intake (as a % of daily energy) of 1% and 0.5%, stratified by age, and gender. Fig A in S1 File, Hospital Admissions of Acute Myocardial Infarction (AMI), Unstable Angina (UA) and Heart Failure (HF) with a 0.5% reduction in daily energy intake of trans fatty acids intake. Fig B in S1 File Deaths prevented or postponed (DPPs) with a trans fatty acids daily energy intake of 0.5% across all socio-economic quintiles. Strobe Statement A in S1 File. (DOC) [file pone.0132524.s001.doc]

**IMPACT England and Wales Trans Fats (IMPACT_TFA_) extension model text A in S1 file.**

**Mortality counterfactual:**

In order to capture and mimic the decreasing trend of the CVD mortality rates, observed in the last decades, an exponential decay model was fitted to past mortality rates from 1993 to 2007. Exponentials decay models are often used when a quantity (i.e. mortality rates) presents a negative growth at a fixed rate (decay rate):


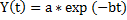


Here
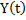
 is the quantity at time
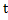
,
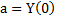
is the initial value before the decay begins and
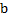
the decay rate. The parameters
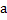
 and
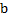
 were estimated using nonlinear least squares fitting method, implemented in the library of MATLAB Curve fitting toolbox.

However, the main objective of fitting the model to the data was to extrapolate the rates to the year 2016, 2020 and 2030. One important feature of the exponential decay models is that
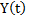
tends to decrease over time asymptotically to zero, that is to say the model would capture how CVD  mortality rates decrease over time avoiding unrealistic negative values. In addition the exponential model would capture the recent flattening of mortality rates observed among some age groups or other complex patterns, if they exist in the data (O’Flaherty et al., 2012).

**Estimating the numbers of death prevented or postponed by changes in trans fats:**

In the regression approach used for trans fats the expected number of deaths from CHD occurring in 2030 were multiplied by the age and gender specific regression coefficient quantifying the change in CHD mortality that would result from the change in trans fats intake levels.

***Mortality fall due to reduction in trans fats in men aged 65-74***

For example, in 2030, 6155 CHD deaths would be expected among men aged 75-84 years in England. The mortality reduction beta coefficients were taken from Mozafarrian et al [1], whilst age and gender specific rates were taken from O’Flaherty et al [2]. A reduction in trans fats intake of 1% of daily energy in the above age and gender group, would have a mortality reduction of 6.0%.

The number of deaths prevented or postponed as a result of this change was then estimated by calculating the mortality reduction factor by the baseline number of expected CHD deaths. Ie:

6155 * 0.060 = 372

This calculation was then repeated

a) for men and women in each age group, and

b) using maximum and minimum values in each group, to generate a sensitivity analysis.

**Probabilistic sensitivity analysis in the IMPACT England and Wales trans fats (IMPACT_TFA_) model.**

Table 1 presents the inputs used in the probabilistic sensitivity analysis of the IMPACT Trans Fats (IMPACT_TFA_) model. The Montecarlo simulation was implemented in Excel using Ersatz (version 1.0 available at [http://www.epigear.com](http://www.epigear.com/)). Ersatz allows repeatedly sample random values from specified distributions for the input variables, and then these values are used to recalculate the model. It then calculates the 95% uncertainty intervals for the output variable (deaths prevented or postponed, LYG and patient numbers). For the IMPACT England Trans Fats (IMPACT_TFA_) model, we calculated the uncertainty intervals based on 5000 iterations taking the 95% uncertainty intervals as the 2.5th and 97.5th percentiles.

**Table A in S1 File: Probability distributions for the IMPACT England and Wales Trans Fats (IMAPCT_TFA_) Model parameters.**

| **Input parameters** | ***Type of distribution and functions*** | **Source** |
| --- | --- | --- |
| **Population and deaths** | | |
| Population counts and CHD deaths stratified by age and sex. | Population counts (assumed no error)  Deaths expected in 2016, 2020 and 2030 had trends in CHD mortality rates in 2007 persisted (***Poisson distribution***) | Office for National Statistics |
| **Estimation of DPP and LYG** | | |
| Beta coefficient | Normal Distribution (mean, SE) | [1,2] |
| Median Survival | Pert distribution (best, min and max: 20%) | [3]  Assumption |
| **Patient numbers calculation** | | |
| Mortality reduction upon baseline patient numbers | Pert distribution (best, min and max; 20%) | Assumption |

**References:**

Mozaffarian D, Katan M, Ascheiro A et al. Trans fatty acids and cardiovascular disease. N Engl J Med 2006;354:1601-13

O’Flaherty M, Flores-Mateo G, Nnoaham K, et al. Potential cardiovascular mortality reductions with stricter food policies in the United Kingdom of Great Britain and Northern Ireland. Bulletin of the World Health Organization 2012;90:522-531

Unal B, Critchley JA, Fidan D et al. Life-years gained from modern cardiological treatments and population risk factor changes in England and Wales, 1981-2000.Am J Public Health. 2005 Jan;95(1):103-8.

**STROBE statement A in S1 file: checklist of items that should be included in reports of observational studies**

**Quantifying the socio-economic benefits of reducing dietary trans fats:**

**modelling study.** Jonathan Pearson-Stuttard et al.

|  | **Item No** | | **Recommendation** | |  | |
| --- | --- | --- | --- | --- | --- | --- |
|  | |  | | **Title and abstract** | |  |
|  | 1 | | (*a*) Indicate the study's design with a commonly used term in the title or the abstract | | Yes | |
|  |  |  | (*b*) Provide in the abstract an informative and balanced summary of what was done and what was found | | Abstract attached | |
|  | |  | | **Introduction** | |  |
| Background/rationale | 2 | | Explain the scientific background and rationale for the investigation being reported | | Background & rationale explained | |
| Objectives | 3 | | State specific objectives, including any pre-specified hypotheses | | Specific objective stated | |
|  | |  | | **Methods** | |  |
| Study design | 4 | | Present key elements of study design early in the paper | | Key elements presented | |
| Setting | 5 | | Describe the setting, locations, and relevant dates, including periods of recruitment, exposure, follow-up, and data collection | | Settings locations and dates specified | |
| Participants | 6 | | (*a*) *Cohort study*? Give the eligibility criteria, and the sources and methods of selection of participants. Describe methods of follow-up *Case-control study*? Give the eligibility criteria, and the sources and methods of case ascertainment and control selection. Give the rationale for the choice of cases and controls  *Cross sectional study*? Give the eligibility criteria, and the sources and methods of selection of participants | | NA  **Cross sectional study.**  eligibility criteria, sources and methods of selection of cases clearly specified. | |
|  |  |  | (*b*) *Cohort study*? For matched studies, give matching criteria and number of exposed and unexposed *Case-control study*? For matched studies, give matching criteria and the number of controls per case | | NA | |
| Variables | 7 | | Clearly define all outcomes,  exposures, predictors, potential confounders, and effect modifiers. Give diagnostic criteria, if applicable | | Outcomes, exposures, predictors, potential confounders, and effect modifiers clearly described. | |
| Data sources/ measurement | 8* | | For each variable of interest, give sources of data and details of methods of assessment (measurement). Describe comparability of assessment methods if there is more than one group | | Diagnostic criteria based on ICD codes. Sources for effect of trans fats given and described in detail.. | |
| Bias | 9 | | Describe any efforts to address potential sources of bias | | Age adjustment and stratification by socio-economic circumstances detailed. | |
| Study size | 10 | | Explain how the study size was arrived at | | NA | |
| Quantitative variables | 11 | | Explain how quantitative variables were handled in the analyses. If applicable, describe which groupings were chosen and why | | Methods described in detail | |
| Statistical methods | 12 | | (*a*) Describe all statistical methods, including those used to control for confounding | | Statistical methods described in detail | |
|  |  |  | (*b*) Describe any methods used to examine subgroups and interactions | | Subgroup analyses detailed | |
|  |  |  | (*c*) Explain how missing data were addressed | | NA | |
|  |  |  | (*d*) *Cohort study*? If applicable, explain how loss to follow-up was addressed *Case-control study*? If applicable, explain how matching of cases and controls was addressed  *Cross sectional study*?If applicable, describe analytical methods taking account of sampling strategy | | NA | |
|  |  |  | (*e*) Describe any sensitivity analyses | | NA | |
|  | |  | | **Results** | |  |
| Participants | 13* | | (*a*) Report numbers of individuals at each stage of study? eg numbers potentially eligible, examined for eligibility, confirmed eligible, included in the study, completing follow-up, and analysed | | Reported | |
|  |  |  | (*b*) Give reasons for non-participation at each stage | | NA | |
|  |  |  | (*c*) Consider use of a flow diagram | | NA | |
| Descriptive data | 14* | | (*a*)Give characteristics of study participants (eg demographic, clinical, social) and information on exposures and potential confounders | | Details provided | |
|  |  |  | (*b*) Indicate number of participants with missing data for each variable of interest | | NA | |
|  |  |  | (*c*) *Cohort study*? Summarise follow-up time (eg average and total amount) | | NA | |
| Outcome data | 15* | | *Cohort study*? Report numbers of outcome events or summary measures over time | | NA | |
|  |  |  | *Case-control study?* Report numbers in each exposure category, or summary measures of exposure | | NA | |
|  |  |  | *Cross sectional study?* Report numbers of outcome events or summary measures | | Events detailed | |
| Main results | 16 | | (*a*) Report the numbers of individuals at each stage of the study? eg numbers potentially eligible, examined for eligibility, confirmed eligible, included in the study, completing follow-up, and analysed | | Numbers detailed | |
|  |  |  | (*b*) Give reasons for non-participation at each stage | | NA | |
|  |  |  | (*c*) Consider use of a flow diagram | | NA | |
| Other analyses | 17 | | Report other analyses done? eg analyses of subgroups and interactions, and sensitivity analyses | | Sub-group analyses and comparisons detailed | |
|  | |  | | **Discussion** | |  |
| Key results | 18 | | Summarise key results with reference to study objectives | | Key results summarised.  Reflect objectives. | |
| Limitations | 19 | | Discuss limitations of the study, taking into account sources of potential bias or imprecision. Discuss both direction and magnitude of any potential bias | | Limitations and potential biases discussed in detail | |
| Interpretation | 20 | | Give a cautious overall interpretation of results considering objectives, limitations, multiplicity of analyses, results from similar studies, and other relevant evidence | | Cautious throughout. | |
| Generalisability | 21 | | Discuss the generalisability (external validity) of the study results | | Generalisability briefly discussed. | |
|  | |  | | **Other information** | |  |
| Funding | 22 | | Give the source of funding and the role of the funders for the present study and, if applicable, for the original study on which the present article is based | | Funding sources detailed | |

**Fig. A in S1 File. Hospital Admissions of Acute Myocardial Infarction (AMI), Unstable Angina (UA) and Heart Failure (HF) with a 0.5% reduction in daily energy intake of trans fatty acids intake.** 1a Male, 1b Female. Hospital admissions by age. *Data source: Hospital Episode Statistics*

*Ai)*

*
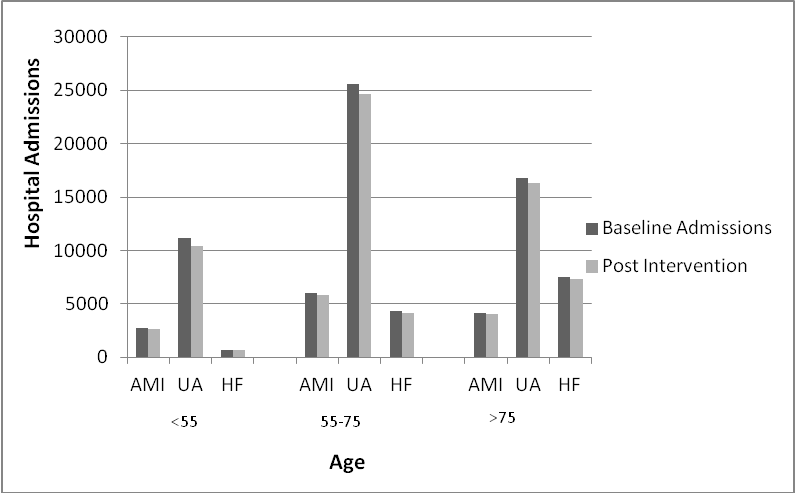
*

*Aii)*

*
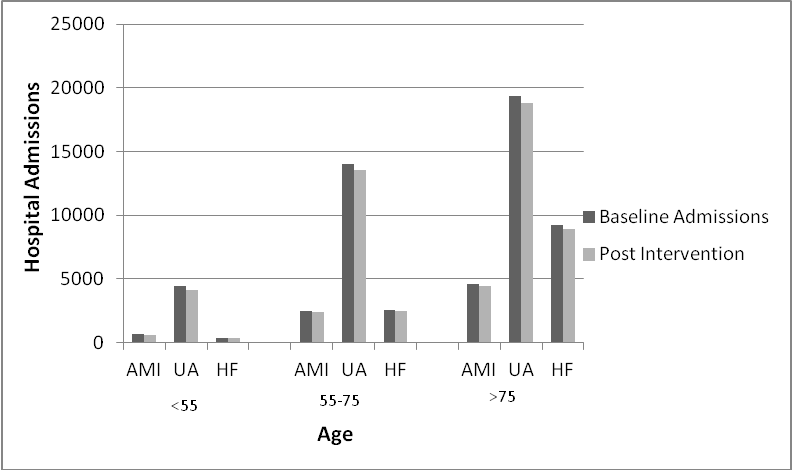
*

**Fig. B in S1 File. Deaths prevented or postponed (DPPs) with a trans fatty acids daily energy intake of 0.5% across all socio-economic quintiles.** DPPs by age, gender and socio-economic circumstance (SEC) quintile. *Data source: Hospital Episode Statistics*

*
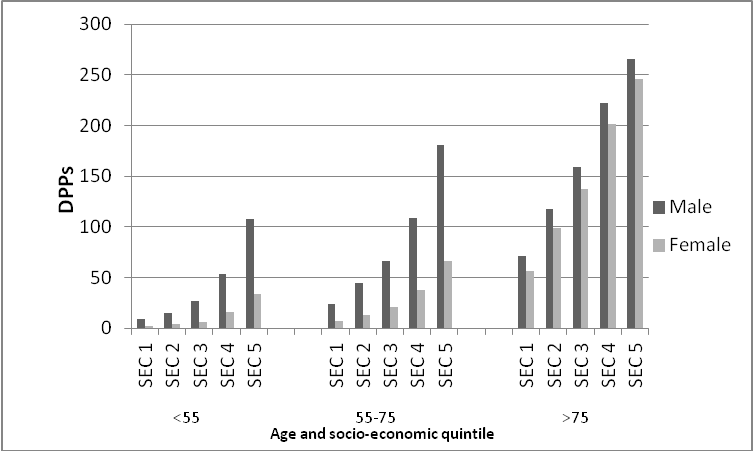
*

***Table B in S1 File – Trans fatty acids intake (as a % of daily energy) by socio-economic circumstance (SEC) quintile.*** *Figures used in TFA_SEC2_. Data Source: Adapted from Low Income Diet and Nutrition Survey [7]*

|  | **SEC 1** | **SEC 2** | **SEC 3** | **SEC 4** | **SEC 5** |
| --- | --- | --- | --- | --- | --- |
| **Trans fats intake** | 0.75% | 0.87% | 1% | 1.25% | 1.50% |

***Table C in S1 file – Mortality reduction factors for reduction in Trans fats intake (as a % of daily energy) of 1% and 0.5%, stratified by age, and gender.*** *Data source: Adapted from Lewington et al [30].*

| **Men** | **0.5% reduction** | **1% reduction** |
| --- | --- | --- |
| **25 - 34** | 0.082 | 0.163 |
| **35 - 44** | 0.082 | 0.163 |
| **45 - 54** | 0.058 | 0.115 |
| **55 - 64** | 0.041 | 0.082 |
| **65 - 74** | 0.030 | 0.060 |
| **75-84** | 0.029 | 0.058 |
| **85+** | 0.028 | 0.056 |
| **Women** |  |  |
| **25 - 34** | 0.082 | 0.163 |
| **35 - 44** | 0.082 | 0.163 |
| **45 - 54** | 0.058 | 0.115 |
| **55 - 64** | 0.041 | 0.082 |
| **65 - 74** | 0.030 | 0.060 |
| **75-84** | 0.029 | 0.058 |
| **85+** | 0.028 | 0.056 |
